# The Practical Value of Chemistry in Dentistry

**Published:** 1899-01

**Authors:** H. C. Wetmore

**Affiliations:** St. John, N. B


THE
AMERICAN JOURNAL
—OF—
DENTAL SCIENCE.

VOL. XXXII. BALTIMORE, JANUARY, 1899. No. 9.

### Selections.

ARTICLE I.

### THE PRACTICAL VALUE OF CHEMISTRY IN DENTISTRY.

H. C. WETMORE, D. D. S., ST. JOHN, N. B,

Read before the Maritime Dental Association, Sept. 1898.

MR. PRESIDENT:—It was not without certain feelings of reluctance that a few weeks ago I consented to write a paper for this joint meeting of the Dental Societies, caused by consciousness of my own inability to treat my topic in a comprehensive and scientific manner, as well as by a knowledge that lack of idle time would render it difficult for me to give any subject proper consideration.

Events which had transpired during the last two or three years, as well as certain experiences which it has been my lot to encounter as a member of an examining board, have been the means of firmly impressing upon my mind that certain of the subjects found on the recognized "list of studies" of our colleges do not apparently receive that attention and consideration at the hands of the teachers, and consequently of the pupils, which they would seem to merit.

Probably if a class of matriculants were given the curriculum of studies required to be taught by a Dental College, and asked to indicate which to them appeared as of the most and which of least importance, whatever might be their opinion as to the former, there can hardly be any doubt that Chemistry would almost invariably be named as the latter.

For a study so fundamental to almost every branch of dental science, to me it would appear that too great a prominence could hardly be given it by those engaged in its teaching, or that too much time and labor could be employed on it by those engaged in its study. If we are to draw our conclusions from the evidence at our disposal, we should conclude that certain of the colleges, supposedly reputable, either make their teachings of it of a very meagre and superficial nature, or they admit to their classes students of insufficient mental calibre to receive and digest such instruction as is imparted. Possibly the error may lie in both directions.

To be more explicit: Were matters otherwise, we would hardly expect a graduate of a "recognized" dental college, who had obtained his degree but a few weeks before, to define "*an element*" as "*the smallest part of matter*," or that another who had also been able to write D. D. S., after his name but a month or so, but from a different college, to define it as, "*that part of a substance in a true or pure state.*"

While to the casual observer Chemistry does not appear to occupy the same prominent position in our daily practice that is accorded other branches of dental science, to the initiated it is of equal importance, in that it is more or less directly connected with them, and in that it forms to a greater or less extent the foundation upon which each of the others is erected. And it is extremely difficult to understand how a college examiner could consistently recommend any student for graduation in Materia Medica, Dental Pathology, Metallurgy or Operative Dentistry, whose knowledge of the very rudiments of chemical science was utterly lacking as in such cases as the above mentioned.

It would make a paper of this nature entirely too cumbersome to attempt to indicate wherein Chemistry has a practical relationship to all the various branches of dental science, even if time permitted, consequently I will confine my remarks to its relation to that particular branch, without which the dental profession could never have begun its existence, namely, its relation to dental caries and to the methods of its prevention and arrest.

Apart from its scientific value, it forms an interesting chapter of historical study to trace the advent of chemical science into the causes furnished by the various writers at different dates for dental caries.

One need revert only about one century into dental history to learn that at that time caries was considered as being the direct result of inflammation, and as bearing a striking resemblance to necrosis of bone, or mortification of soft tissues. It is also of interest to learn that at this period writers were a unit in supposing that the initial stages of caries took place in the *dentine*, and from there the inflammation extended, causing death to all the adjacent parts.

The first divergence from this view was when certain writers maintained that sometimes caries began upon the surface of a tooth, and the inflammatory theory then partook of a dual nature, and caries was described as being of either external or internal origin. Not until the year 1830, or almost in recent times, was this theory questioned; and almost simultaneously Harris of America, Robertson of England, and Regnard of France proved by experiment and clinical experience that it no longer was tenable. A few years later Robertson published a "Treatise on the Human Teeth," showing causes of their decay and means of preservation, in which he advanced the theory that caries was the result of chemical disintegration of the tooth substance, and denied the agency of inflammation. This destruction was accomplished, he contended, by the action of an acid which was generated by decomposition of alimentary particles, or of fluids of the mouth suffered to lodge about the teeth. Three years later Regnard published a somewhat similar work, in which he contended that caries was accomplished by an acid generated by decomposition taking place at the very point where its effects were shown.

We must bear in mind that at the time these authors wrote the best of human thought and inteligence, as well as the deductions from all observations except their own, were diametrically opposed to their theory (a theory which all the labor of intervening years up to the present time has hardly been able to demonstrate). And their writings appear the more remarkable since, at that time, the laws of fermentation were very little known, and they had not means of confirming their suppositions by experiments made either by themselves or by others.

It was curious to notice that once the chemical theory was advanced, how prone many men were to run to the opposite extreme, and assert that if acids caused decay they would necessarily, from their general distribution, act upon all parts of the teeth, instead of attacking only particular parts. Black in his writings, which were founded upon his own experimental results, says: "I may say that the acidity or alkalinity of the general fluids of the mouth or of the food plays but a small part in the case, provided these reactions be not in such degree as to modify materially the act of fermentation taking place in the out-of-the-way points about the teeth. The teeth may decay when the fluids of the mouth are habitually acid, or when they are habitually alkaline. The condition governing the beginning and progress of decay is neither of these, but is dependent directly on the lodgment of substances at particular points and their fermentation with the production of an acid. It is in this manner that caries has its beginning, and its progress is maintained by the continuance of this act of fermentation."

Perhaps no one person has thrown as much light on the processes of fermentation as exhibited in the early stages of caries as Prof. Miller, of Berlin. He, in a very extensive series of experiments, most carefully planned and executed, was enabled to establish truths which heretofore could be held but as conjectures.

In the first of these he established the initial stage in the chemical theory, namely, that saliva, to which has been added a sterilized organic body as starch or sugar, will become acid in four of five hours, indicating the splendid opportunity organisms of the saliva possess in the mouth during certain periods of the day or night, particularly the latter, to uninterruptedly propagate and exert their destructive influence on the surrounding teeth. It was already known that an active unorganized ferment ptyalin already existed in the saliva, but Miller demonstrated the organism producing the acid to be very different in its nature and properties; that it was an organized ferment, and was perfectly capable of sustaining and reproducing itself in a suitable pabulum. To determine the nature of the acid produced by this process of fermentation was a problem of no great difficulty, and a few experiments proved what was already conjectured, that it was no more than the ordinary ferment, lactic acid.

More important to the development of dental science was the demonstrating of artificially produced decalcification and caries in a sound tooth, by placing it in a suitable pabulum: a solution of beef extract and cane-sugar, which after being sterilized was infected with the fungus already referred to, thus proving the correctness of the chemical theory. With these agents, fungi, and a suitable soil on which it may feed and luxuriate, aided indirectly by a uniform temperature, moisture and free oxygen, it requires but a very superficial knowledge of the nature and habits of fungi to comprehend how an acid, their product, will almost invariably be found, not necessarily in the entire mouth, that may be alkaline, but in spaces adapted for the lodgment of food particles, in the fissures, sulci and inter-dental spaces of the teeth.

We do not wish to be understood as stating that lactic acid is the only acid, or even the only destructive acid, of the oral cavity. The acidity of the mucus secretion is well known, and while it does not possess decalcifying properties to the same degree as lactic acid, it assists very materially the destructive influence of lactic acid in cavities bordering upon the gum margin, if indeed it is not the sole cause of decalcification in such cases.

Other fungi than the one we have been considering are also to be found in the secretions of the mouth, Black having recognized as many as twenty-two varieties, and whatever their influence may be, it appears evident that so far as caries is concerned they are not particularly harmful.

Having established the fact that caries is to a certain extent the direct result of the action of ferment acids on the teeth, it becomes us next to ascertain how we can scientifically most effectually combat their influence, or prevent their production; and secondly, how we can prevent already decalcified dentine from further or complete destruction.

By thorough, repeated and systematic cleansing of the oral cavity and teeth we may so diminish the amount of fermentable substance as to very much lessen the production of acids by fermentation. This is so evident that comment is unnecessary. By a repeated application of alkaline substances one may to a very considerable extent counteract the decalcifying action of acids before extensive loss has been sustained. While, by a proper and intelligent use of antiseptics, we may destroy the organisms themselves, or at least render them inactive. It is this method which is specially applicable in the stage or caries following decalcification, and the one which at present we are considering.

Even here we must constantly bear in mind that a previous thorough cleansing is indispensable. There is no solution, alkaline or antiseptic, applicable to the human mouth which will penetrate between the teeth, or to the bottom of fissures or cavities, when these are filled with food or other debris, in sufficient quantity to have an appreciable effect upon either acid or organism; so that before any washes can be expected to accomplish anything whatever, must first come a thorough use of tooth brush, tooth pick and floss silk. To furnish formulæ is not our object in this paper, but the primary object in writing any medical prescription is to first ascertain the cause of the ailment, get at the first principles of the disease and combat and, if possible, destroy them. Reasoning from what we know of the more prominent causes of caries, an antiseptic, effective in its workings, yet of a nature which would cause no injury to the delicate tissues of the mouth, with which it must be brought in contact; and if possible, of alkaline reaction, would appear to combine the properties essential to the case.

Where caries has progressed it is naturally next to impossible, in many cases, in the preparation of cavities to remove all of the affected dentine, and in such cases it is desirable, as far as possible, to insert fillings which are not only antiseptic at the time of insertion, but which will continue to permanently exercise antiseptic influence. Of the filling materials most commonly used most are antiseptic at the time of insertion, but gradually lose their antiseptic properties.

Old fillings of phosphates are found to possess but a slight antiseptic power.

Old fillings of gold and tin combined have also a slight antiseptic power, and gold amalgam fillings, when not containing copper, have very much less, if in fact they can be said to have any at all. But if they contain copper the result is very different, it has then a very marked continuous antiseptic effect; the salts permeating the dentine of the teeth so filled, and rendering it (the discolored dentine) decidedly more powerfully antiseptic than any of the other filling materials. It, however, has its disadvantages. But what we would like to impress is the importance of a knowledge of the chemical effect of the ingredients, upon organisms and truth structure, of the amalgams we use, and make our selections to meet the requirements of our individual cases, even though some other varieties may be represented as being "just as good," and can be furnished by a rival house at a somewhat lesser cost. Any reliable house ought to be willing to furnish the formulæ of their amalgams if they expect intelligent dentists to employ them in their practice.

We will refer to but one other instance of the practical value of chemical science to the dentist in his professional duties, the chemico electric effect of the various metals more commonly placed in the oral cavity in either operative or prosthetic operations. Electricity at the present time is occupying such an important position in the various departments of dentistry that it is difficult to comprehend how a course of dental study could approach completeness without including a fair treatment of its more common principles and application.

Leaving to inventors its usefulness as a power furnishing agent, either for the revolving of a wheel or driving a medicine through a tissue, we will confine our attention to some of the simpler phenomena of galvanism as they come to our attention in our daily operations.

We aim to be a progressive profession, and if we are to continue a recognized profession we must continue to be as progressive as our brothers in other walks of life, and as progressive as the age in which we live. We have all observed phenomena in our operations when both gold and amalgam have been employed, which, without some knowledge of the laws of galvanism, would be rendered impossible of explanation. Observation teaches us that normal tooth structure is a non-conductor of galvanic current, while with dentine badly deficient of lime salts, or decalcified by the aid of oral acids, the conditions become the reverse and render it highly conductive and electro-positive as compared with either gold or amalgam. It would be as unreasonable to expect no electric disturbance when these metals were used in proximity to dentine in such cases, as it would be to expect that a copper or carbon plate immersed in a cell containing all the other elements for a galvanic current, should produce no result. In the case of a gold or amalgam filling loosened, the effect is frequently so pernicious that after its removal further operations must for the time being be suspended, allowing the tooth to regain to some extent its normal status.

Kirk says that even in the selection of metal for clasps too great care cannot be exercised, that either silver or gold clasps on a plate of the same metal are apt to be much more injurious than when they are made of a metal different from the plate. That in the first instance the tooth forms the positive and the plate the negative elements of the battery, while in the latter the tooth is left entirely out of the circuit, and what galvanism does exist is simply between the metals of the plate and clasp. It is also a recognized fact that when it becomes necessary to insert a filling under a clasp, an amalgam filling will prove more effective in arresting the progress of caries than an equally well manipulated gold. In most mouths under these conditions, electrical action would manifest itself, and the method which would leave the tooth out of the circuit, as the gold and amalgam would do, would most assuredly protect it from the evil influence of galvanic action. Electro chemical action in the oral cavity will be found to be greater during the night than the day, on account of the increased acidity of oral secretions during that time. Apart from this greater opportunity for the acids of fermentation to accumulate, the mucus secretion which is slightly acid in reaction, is greater during the night, while the neutralizing effect of the salivary flow, which normally is alkaline, is practically lost, as the amount of saliva secreted at that time is greatly diminished. Ample reasons, it would appear, are thus furnished for the careful removal of all clasp dentines, or other metal appliances, when practicable, before retiring.

Other instances might easily be added, but enough, we consider, has been submitted, to warrant us in concluding that a course of dental chemistry, whatever it may include, would be far from complete if it did not embrace the simpler laws and principles of galvanism; notwithstanding this, the evidence appears to prove the contrary; were it otherwise we would hardly expect a man fresh from college, with the teachings of three years received still clear in his memory, to furnish the following comprehensive description of a galvanic cell: "*A galvanic cell is composed of two substances; one is copper, the other is the other.*" —*Dominion Dental Journal.*

